# New species of *Solanum* (Solanaceae) from  Peru and Ecuador

**DOI:** 10.3897/phytokeys.1.659

**Published:** 2010-11-01

**Authors:** Sandra Knapp

**Affiliations:** Department of Botany, The Natural History Museum, Cromwell Road, London SW7 5BD, United Kingdom; Department of Botany, The Natural History Museum, Cromwell Road, London SW7 5BD, United Kingdom

**Keywords:** Amotape-Huancabamba zone, Andes, Ecuador, endemism, conservation, nightshades, Peru, *Solanum*

## Abstract

Three new species of “non-spiny" Solanum are described from Peru and Ecuador, and a revised description for Solanum verecundum M. Nee is presented. Solanum kulliwaita S. Knapp, **sp. nov.** (Dulcamaroid clade) is endemic to the Department of Cuzco in southern Peru, and is most similar to the recently described Solanum sanchez-vegae S. Knapp of northern Peru. Solanum dillonii S. Knapp, **sp. nov.** (Brevantherum clade) is found in southern Ecuador and northern Peru in the Amotape-Huancabamba phytogeographic zone, and is morphologically similar to the widespread Solanum riparium Ruiz & Pav. Solanum oxapampense S. Knapp, **sp. nov.**, (also of the Brevantherum clade) is endemic to the Oxapampa region (Department of Pasco) of central Peru, and is similar to and segregated from Solanum verecundum M. Nee of Peru and Ecuador. Complete descriptions, distributions and preliminary conservation assessments of all new species are given.

## Introduction

Solanum L., with ca. 1500 species, is the largest genus in the Solanaceae and one of the ten most species-rich genera of flowering plants (Frodin 2004). The highest species diversity in the genus occurs in South America, and is concentrated in the Andes (Knapp 2002). As part of the collaborative project PBI Solanum: a world--wide treatment (see Knapp et al. 2004, http://www.solanaceaesource.org), descriptions of all species of Solanum are being provided on-line. This intensity of work in the genus by a large number of collaborators, along with the massive increase in specimens available from the Andean regions of South America and intensive work in the undetermined collections of herbaria worldwide has meant that species limits are being re-evaluated using modern methods (e.g., Spooner et al. 2008, Ames and Spooner 2010) and many new taxa are being discovered, both in the field and in herbaria (see Chiarini 2004, Knapp 2005, Peralta et al. 2005, Anderson et al. 2006, Granados-Tochoy and Orozco 2006, Nee et al. 2006, Granados-Tochoy et al. 2007, Knapp 2007, 2008, Knapp and Nee 2009, Stern and Bohs 2009, Tepe and Bohs 2009, Knapp 2010). In addition, the intensive global monographic project, in conjunction with a commitment by the botanical community to achieving Targets 1 (a global plant species checklist) and 2 (preliminary conservation assessments for all known plant species) of the *Global Strategy for Plant Conservation* (GSPC; Secretariat of the CBD 2002), means that recognition and description of endemic or near endemic taxa or those facing a significant conservation threat is particularly timely.

Recent intensive collecting in Peru, coupled with targeted collecting by members of the PBI project team and work in many herbaria has uncovered several new species from Peru and adjacent Ecuador and necessitated the revision of the circumscription of Solanum verecundum M. Nee from which one of these new taxa is segregated. Two of these are endemic to Peru and one to the recently defined highly diverse Amotape-Huancabamba phytogeographic zone (Weigend 2002, Stern et al. 2009) straddling the border of Peru and Ecuador. All of these taxa have been assessed for conservation status using the ArcGIS software described in Moat (2007) which uses a combination of extent of occurrence (EOO), a measure of geographic spread as a polygon, and AOO (area of occurrence), a measure of distribution based on number of occurrences. For calculation of the AOO I have used both a cell size of 0.04 km2 as recommended by Moat (2007) and of 2 km2 as recommended by IUCN (2001). Coordinates are presented in square brackets if calculated from maps; otherwise they are given as written on specimen labels.

## Taxonomic treatments

### Dulcamaroid clade

Members of the Dulcamaroid clade (sensu Bohs 2005, Weese and Bohs 2007) have terminal and usually highly branched inflorescences, pedicels arising from small pegs on the inflorescence rachis and a vine-like habit. The clade is sister to the black nightshades (Morelloids, including members of Solanum section Solanum), and contains 50 species of woody and semi-woody vines and lax shrubs. Four of these species are from Eurasia (including the widespread weed Solanum dulcamara L.), four are from North America (including Mexico) and the rest are from Central and South America, with centers of diversity in the Andes and southeastern Brazil. 

#### Solanum kulliwaita

S. Knapp sp. nov.

urn:lsid:ipni.org:names:77107687-1

Fig. 1 

##### Latin

*Species nova* Solano sanchez-vegae *mihi similis, sed foliis ad apicem acuminatis, inflorescentibus et floribus glandularibus, trichomatibus uniseriatis simplicibus differt.*

##### Type.

**Peru:** Cusco: Prov. La Convención, Dist. Ocobamba, Mesa Pelada, 12°54.13S, 72°37.06W, 2613 m, 23 March 2004, L. Valenzuela, E. Suclli & G. Calatayud 3163 (holotype: USM!; isotypes: AMAZ, CUZ, MO!, MOL, NY! [NY00824906]).

##### Description.

Woody vine or scandent shrub, height unknown, the branches arching. Stems sparsely pubescent with simple uniseriate multicellular trichomes 0.5–1 mm long, glabrescent, slightly winged from the decurrent leaf bases; new growth pubescent with simple or occasionally branched uniseriate trichomes 0.5–1 mm. Bark of older stems dark reddish brown, shiny. Sympodial units plurifoliate. Leaves simple, (2-)3.5–8.5 cm long, 1–3 cm wide, narrowly elliptic to lanceolate, slightly fleshy, the upper surfaces sparsely pubescent with simple or occasionally furcate or branched trichomes on the lamina, more densely pubescent on the midvein, the lower surfaces glabrous or with a few scattered simple uniseriate trichomes along the midvein; primary veins 7–9 pairs, often drying blackish brown; base acute to attenuate; margins entire, sometimes revolute, densely pubescent in the basal quarter to third with simple trichomes extending from the petiole; apex acute; petioles 0.7–2 cm long, densely pubescent along the adaxial groove with golden simple or occasionally furcate uniseriate trichomes, not apparently twining. Inflorescences terminal or appearing lateral, 9–11 cm long, 3–5 times branched, with 10–20 flowers, densely pubescent with simple uniseriate trichomes mostly 0.3–0.5 mm long, some longer and to 1 mm, purple in live plants and retaining pigmentation in dried material, the cells of the trichomes small and weak-walled, usually collapsing and tangled, the lateral cell walls dark-pigmented, the terminal cells spheroidal and apparently glandular; peduncle 1.5–3.5 cm long; pedicels 1–1.2 cm long, ca. 0.5 mm in diameter at the base, ca. 1 mm in diameter at the apex, slender, erect to nodding, densely pubescent like the inflorescence axes, articulated at the base and inserted into a short sleeve or above the base and leaving a peg ca. 2 mm long; pedicel scars irregularly spaced 0.5–5 mm apart, usually grouped. Buds ellipsoid, the corolla strongly exserted from the calyx tube before anthesis. Flowers all perfect, 5-merous. Calyx tube 2–2.5 mm long, cup-shaped, narrowing gradually to the pedicel, the lobes 2.5–3.5 mm long, the lower portion broadly deltate, the distal part an apiculate tip to 2 mm long, densely pubescent with simple uniseriate trichomes like those of the inflorescence axes abaxially, these apparently glandular, the adaxial surface glabrous. Corolla 2.3–2.5 cm in diameter, purple, stellate, lobed 2/3 to ¾ of the way to the base, the lobes 9–12 mm long, 4–5 mm wide, spreading, the tips and margins densely pubescent on the abaxial surface with weak, collapsing simple uniseriate trichomes like those of the inflorescence, but smaller and not apparently glandular. Filament tube minute, the free portion of the filaments 1–2 mm long, glabrous; anthers 3.5–4.5 mm long, 1–1.5 mm wide, ellipsoidal, loosely connivent, yellow, poricidal at the tips, the pores lengthening to slits with age. Ovary glabrous; style 7–8 mm long, glabrous; stigma capitate, the surface minutely papillose. Fruit a globose berry, ca. 1 cm in diameter (immature?), black when ripe, the pericarp thin, not shiny, glabrous; fruiting pedicels 1.5–1.7 cm long, ca. 1.5 mm in diameter at the base, woody, more or less nodding. Seeds not known.

**Figure 1.**
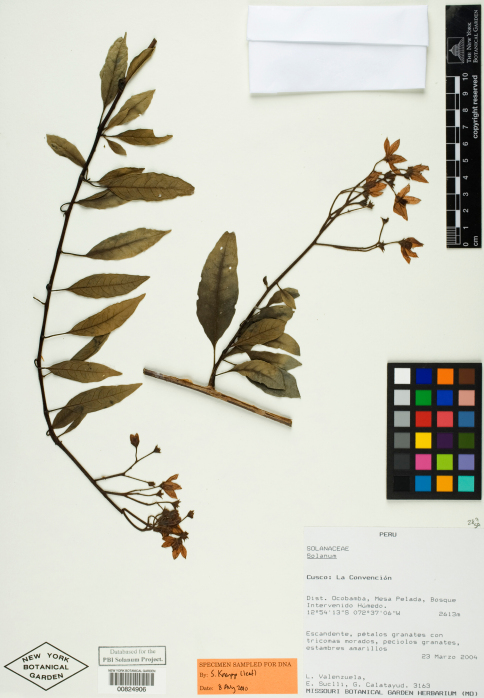
Isotype specimen of Solanum kulliwaita S. Knapp. (Valenzuela et al. 3163, NY [NY00824906]). Specimen image reproduced with the permission of The C. V. Starr Virtual Herbarium of The New York Botanical Garden (http://sciweb.nybg.org/science2/VirtualHerbarium.asp).

##### Distribution.

**Figure 2.**
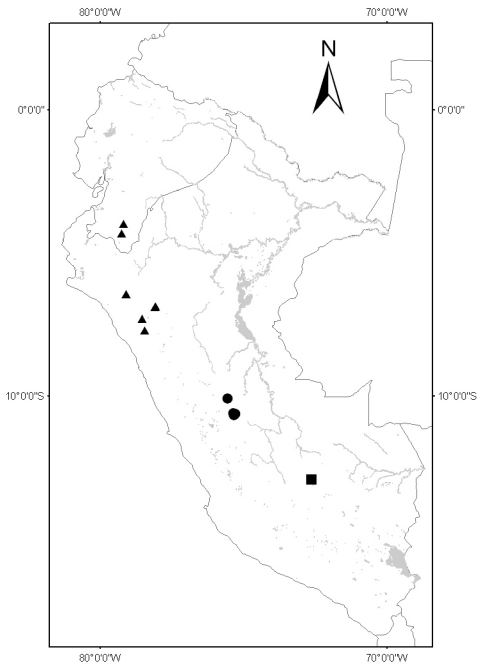
Distribution of Solanum kulliwaita (circles), Solanum dillonii (triangles) and Solanum oxapampense (squares).

Endemic to the valley of the Río Urubamba in the Department of Cusco in southern Peru (Fig. 2).

##### Ecology.

Both collections are from montane cloud forests of the eastern slopes downriver from Machu Picchu in a locality broadly known as Mesa Pelada, from 2400–2600 m.

##### Etymology.

Named for the flower and trichome colour – kulli = purple; waita = flower in Quechua.

##### Preliminary conservation status.

Known only from two collections in a very narrow geographical area outside any protected area, Solanum kulliwaita is assessed as Data Deficient for EOO and AOO (due to there not being 3 collection points with which to calculate the polygon: Moat, 2007); using the cell size of 2 km2, however, is assessed as Critically Endangered (IUCN 2001) with an AOO of 8.

##### Specimen examined.

**Peru:** Cusco: Prov. La Convención, Dist. Maranura, Mesapelada, 12°54.33S, 72°37.06W, 2450 m, 19 April 2004, W. Galiano, E. Suclli, P. Núñez, A. Rodriguez & V. Chama 6137 (CUZ, MO, NY! [NY00824933], USM).

##### Discussion.

Solanum kulliwaita is most similar morphologically to Solanum sanchez-vegae S. Knapp from northern Peru; both species have large, purple flowers and simple leaves. It can be distinguished from the latter species by its leaves that are glabrous beneath and with a dense covering of uniseriate trichomes on the adaxial surface of the petiole, the ciliate lower leaf margins and the distinctive uniseriate glandular trichomes of the inflorescence. Solanum sanchez-vegae has larger, fleshier leaves with loose dendritic trichomes and the trichomes of the inflorescence are both non-glandular and dendritic. The inflorescence trichomes of Solanum kulliwaita are unusual in members of the Dulcamaroid clade in having three globular cells at the apex and in drying purple (and being purple in live plants, fide Valenzuela et al. 3163). Trichomes on the rest of the plant (i.e., leaves and stems) are not glandular.

### Brevantherum clade

Members of the Brevantherum clade (sensu Bohs 2005, Weese and Bohs 2007) are trees, shrubs and herbs with stellate or modified stellate (see Roe 1971, Stern and Bohs 2009) trichomes and oblong to ellipsoid (never tapered) anthers. The two new species described here belong to the group previously recognised as section Brevantherum Seithe (Roe 1967, 1972), distinguished by plurifoliate, dichasial sympodial units and erect, terminal, many-branched inflorescences.

#### Solanum dillonii

S. Knapp sp. nov.

urn:lsid:ipni.org:names:77107688-1

Fig. 3 

##### Latin

*Species nova* Solano ripario *Ruiz & Pav. similis, sed trichomatibus multangulatis vel echinatis longistipitatis, foliis ad basibus acutis, floribus violaceis, differt.*

##### Type.

**Peru:** Cajamarca: Prov. Celendin, on road from Celendin to Balsas, east of pass on descent to Balsas, 2002 m, 6°52.13S, 78°30.91W, 12 December 2007, S. Stern, E. Tepe, S. Leiva & M. Zapata 119 (holotype: USM!; isotypes: BM! [BM001016881], HAO†, NY! [NY 00986687], UT!).

##### Description.

Shrub or small tree, 4–8 m tall, branching in the upper part of the stems. Stems densely pubescent with multangulate to echinoid trichomes on multiseriate stalks 0.5–1 mm long, the rays > 12, 0.2–0.3 mm long, glabrescent; new growth densely pubescent with multangulate to echinoid trichomes on multiseriate stalks 0.5–1 mm long like those of the stems, greyish white. Bark of older stems reddish brown. Sympodial units plurifoliate, the branching dichasial. Leaves simple, 12–30 cm long, 4.5–16 cm wide, elliptic to broadly elliptic, discolorous, the upper surfaces evenly and moderately pubescent with 1–3-rayed sessile stellate trichomes, the rays 0.3–1 mm long, the trichome bases bulbous, the lamina clearly visible, the lower surfaces densely pubescent with of multangulate to echinoid trichomes on multiseriate stalks 0.5–1.5 mm long, the rays 10–16, to 1 mm long, these mixed with porrect-stellate trichomes with 8–10 rays on multiseriate stalks to 1 mm long, and sessile echinoid trichomes with weak rays to 0.3 mm long, the lamina not visible; primary veins 9–11 pairs, the veins drying yellowish green above, not visible beneath; base acute; margins entire, plane; apex acute; petioles 1.5–3 cm long, densely pubescent with multangulate to echinoid trichomes like those of the stems and leaf undersurfaces. Inflorescences terminal, 15–20 cm long, many times branched, with 100+ flowers, densely pubescent with multangulate trichomes of many sizes, the largest to 0.l8 mm in diameter, on multiseriate stalks to 1.5 mm, some smaller and sessile;  peduncle 7–10 cm long; pedicels 7–9 mm long, ca. 2 mm in diameter at the base, 2.5–3 mm in diameter at the apex, stout, nodding at anthesis, densely pubescent like the inflorescence axes, articulated at the base; pedicel scars closely spaced ca. 1 mm apart. Buds globose, the corolla scarcely exserted from the calyx tube before anthesis. Flowers all perfect, 5-merous. Calyx tube 3–3.5 mm long, cup-shaped, narrowing gradually to the pedicel, the lobes 2.5–3 mm long, deltate, densely pubescent abaxially with multangulate to echinoid trichomes like those of the inflorescence rhachis, these more sessile distally, the adaxial surface sparsely pubescent with sessile echinoid trichomes. Corolla 1.3–1.5 cm in diameter, purple, stellate, lobed ca. ¾ of the way to the base, the lobes 6–7 mm long, 3.5–4.5 mm wide, reflexed or spreading at anthesis, the tips and margins densely pubescent on the abaxial surface with sessile or short-stalked multangulate to echinoid trichomes with >10 rays like those of the inflorescence, the adaxial surface glabrous or with a few echinoid trichomes near the apex on the midvein. Filament tube minute, the free portion of the filaments 2.5–3 mm long, glabrous; anthers 3–3.5 mm long, ca. 1 mm wide, ellipsoidal, loosely connivent, yellow, poricidal at the tips, the pores lengthening to slits with age. Ovary densely pubescent with multangulate trichomes; style 9–9.5 mm long, sparsely pubescent along its entire length with multangulate trichomes with 4–8 rays; stigma clavate, the surface minutely papillose. Fruit a globose berry, 1–1.5 cm in diameter, dark green when ripe, the pericarp thin, not shiny, unevenly pubescent with sessile or short-stalked multangulate trichomes with rays of many varying lengths, the longest rays ca. 1 mm long; fruiting pedicels 1.2–1.5 cm long, ca. 3 mm in diameter at the base, woody, more or less erect. Seeds >200 per berry, 1.5–2 mm long, 1–1.5 mm wide, flattened-reniform, reddish or golden brown, the surfaces minutely pitted.

**Figure 3.**
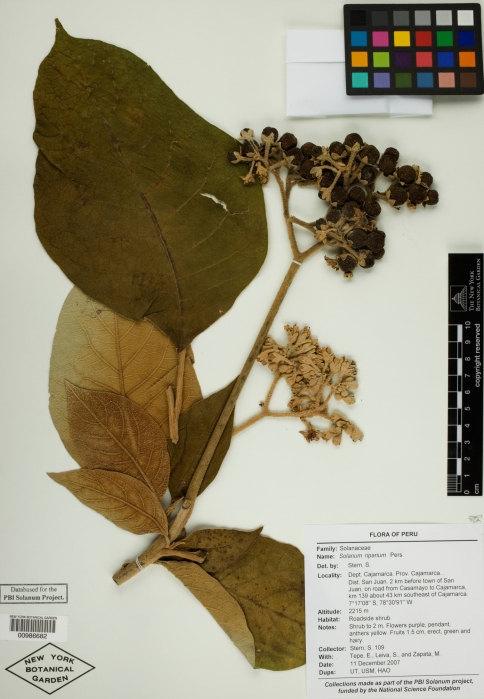
Isotype specimen of Solanum dillonii S. Knapp. (Stern et al. 119, NY [NY00986687]). Specimen image reproduced with the permission of The C. V. Starr Virtual Herbarium of The New York Botanical Garden (http://sciweb.nybg.org/science2/VirtualHerbarium.asp).

##### Distribution.

Southern Ecuador (Prov. Loja) and northern Peru (Dept. Cajamarca), in the Amotape-Huancabamba phytogeographic zone (see Weigend 2002, 2004) (Fig. 2).

##### Ecology.

Tropical moist forest along the western slopes of the Andes and the valley of the Río Marañon, from 1500–2200 m. Often found along roads and small streams in secondary situations.

##### Etymology.

Named in honor of Michael O. Dillon, recently retired from the Field Museum of Natural History in Chicago, who has devoted much time and energy to the understanding of the flora of northern Peru and whose efforts collecting in the Monteseco region helped define the extent of this species' distribution.

##### Preliminary conservation status.

Solanum dillonii is relatively widely distributed in the Amotape-Huancabamba region, and has an EOO of 22,492 km2, giving a status of possible Near Threatened, and an AOO (with cell size of 0.04 km 2) of 10,541 km2, giving a status of Least Concern (IUCN 2001). Using a cell size of 2 km2 gives an AOO of 28 km2, resulting in an assessment of Endangered. Given that the species is of a partially secondary nature, growing in disturbed areas and along roads, I feel the status of possibly Near Threatened is the most realistic assessment for Solanum dillonii, but more collecting with population assessment will help refine this.

##### Specimens examined.

**Ecuador:** Loja: Vilcabamba-Yangana road km 1–3, 1500–1600 m, 4°18'S, 79°14'W, 29 October 1984, P.M. Jørgensen 56267 (AAU, BM [BM001012348], QCA); ca. 7 km E of Catamayo on the road to Loja, ca. 1700 m, 3°28'S, 79°10'W, 6 February 1984, S. Knapp & J. Mallet 6258 (BH, QCA, NY [NY00829072], US). **Peru:** Cajamarca: Prov. Santa Cruz, Dist. Catache, upper Río Zaña valley, ca. 5 km above Monte Seco on path to Chorro Blanco, 1500–2000 m, [6°52'S, 79°05' W], 16–18 March 1986, M.O. Dillon, A. Sagástegui A., D. Dillon, P. Alcorn, J. Santisteban, S. Leiva, C. Téllez & M. Guzmán 4379 (BM [BM000849416], F [F-1993682], NY [NY00829133]); Prov. Santa Cruz, ca. 3 km por aire ENE Monteseco, 1800 m, [6°52'S, 79°05'W], 31 May 1987, J. Santisteban C. & J. Guevara B. 125 (BM, NY [NY00829131]); Prov. Celendin, Marañon River valley, Chachapoyas-Cajamarca road, 1900–2100 m, [6°51'S, 78°04'W], 28 May 1984, D.N. Smith & J. Cabanillas 7257 (MO [MO-5294201], NY [NY00829129]); Prov. Cajamarca, Dist. San Juan, 2 km before town of San Juan, on road from Casamayo to Cajamarca, km 139 about 143 km southeast of Cajamarca, 2215 m, 7°17'08"S, 78°30'91"W, S. Stern, E. Tepe, S. Leiva & M. Zapata 109 (BM [BM001016880], HAO†, NY [NY00986682], USM, UT). La Libertad: Prov. Otuzco, alrededores de Huaranchal, 2140 m, [7°41'21"S, 78°26'51"W], 6 February 1999, A. Sagástegui, S. Leiva & V. Quipuscoa 11608 (BM [BM000935134]).

##### Discussion.

Solanum dillonii is superficially similar to the widespread Solanum riparium Ruiz & Pav. and to the more southerly Solanum conglobatum Dunal. It differs from Solanum riparium in its violet flowers, acute leaf bases and pubescence; both stem and leaf trichomes of Solanum dillonii are very long-stalked and multangulate, while those of Solanum riparium are sessile and tend to have more rays (tending to echinoid sensu Roe 1971). Both species have some porrect-stellate trichomes on the lower leaf surfaces. Solanum riparium occurs in a wide variety of tropical and premontane forests of the eastern Andean slopes, and Solanum dillonii, while to some extent (in the Río Marañon valley) sympatric with it, is a plant of the moist to dry forests of the western Andean slopes and inter-Andean valleys in the Amotape-Huancabamba phytogeographic zone (Weigend 2002, 2004). Solanum dillonii is the species referred to as “Solanum erianthum vel. aff." in the checklist of the Monte Seco forest fragment (Sagástegui and Dillon 1991). Solanum dillonii has also been misidentified as Solanum conglobatum, a species of dry forests from southern Peru and Bolivia. It is similar to Solanum conglobatum, with few-rayed trichomes on the upper leaf surfaces and dense abaxial leaf pubescence, but differs from that species in having multangulate rather than porrect-stellate trichomes abaxially and in not having an accrescent calyx in fruit.

Solanum dillonii is another species endemic to the Amotape-Huancabamba phytogeographic zone (Weigend 2002, 2004); this region has been highlighted as a center of species richness and endemism in the Geminata clade (see Knapp 2002, Stern et al. 2009) of Solanum. The area is not only home to many endemics, such as Solanum dillonii, but is a zone of considerable overlap between northern and southern taxa.

#### Solanum oxapampense

S. Knapp sp.nov.

urn:lsid:ipni.org:names:77107689-1

Fig. 4 

##### Latin

*Species nova* Solano verecundo *M. Nee similis, sed foliis coriaceis superne nitidibus, subtus valde pubescentibus, trichomatibus peltatis, differt.*

##### Type.

**Peru:** Pasco: Prov. Oxapampa, Oxapampa-Villa Rica road, 7 km from road head, 2120 m, 10°36'S, 75°20'W, 4 January 1984, D.N. Smith & J. Albán 5558 (holotype: USM! [USM-123391]; isotypes: MO! [MO- 5784802], NY! [NY00723838]).

##### Description.

Treelet to small tree, 2.5–9 (–18) m tall, branching in the upper part of the stems. Stems densely pubescent with persistent short-stalked peltate trichomes 0.2–0.4 mm in diameter, the rays 20–30, fused for almost their entire length, the midpoint absent, the trichome center dark reddish brown; new growth densely pubescent with peltate trichomes like those of the stems, drying pale beige. Bark of older stems pale brownish tan from the persistent trichomes. Sympodial units plurifoliate, the branching dichasial. Leaves simple, 6.5–16 cm long, 2–5 cm wide, narrowly elliptic, coriaceous, strongly discolorous, the upper surfaces glabrous and shiny, dark green when fresh, drying dark olive green, the lower surfaces densely pubescent with short-stalked peltate trichomes to 0.5 mm in diameter with >20 rays, subtended by a dense layer of tangled sessile echinoid trichomes ca. 0.1 mm long, the lamina not visible; primary veins 16–20 pairs, deeply impressed above, densely covered by pubescence beneath; base acute; margins entire, revolute; apex acute to acuminate; petioles 0.6–2 cm long, densely pubescent with peltate trichomes like those of the stems and leaf undersurfaces. Inflorescences terminal, 8–15 cm long, many times branched, with 60+ flowers, densely pubescent with peltate trichomes like those of the stems; peduncle 3–6 cm long; pedicels 5–8 mm long, ca. 1.5 mm in diameter at the base, ca.2 mm in diameter at the apex, stout, nodding at anthesis, densely pubescent like the inflorescence axes, articulated at the base; pedicel scars closely and more or less regularly spaced ca. 1 mm apart. Buds globose, the corolla exserted about halfway from the calyx tube just before anthesis. Flowers all perfect, 5-merous. Calyx tube 1.5–2 mm long, cup-shaped, narrowing gradually to the pedicel, the lobes 1.5–2 mm long, deltate, densely pubescent abaxially with peltate trichomes like those of the inflorescence rhachis, the adaxial surface sparsely pubescent with sessile echinoid trichomes. Corolla 1.2–1.5 cm in diameter, white, stellate, lobed ca. ¾ of the way to the base, the lobes 6–7 mm long, 4–4.5 mm wide, reflexed at anthesis, the tips and margins densely pubescent on the abaxial surface with peltate trichomes with >20 rays like those of the inflorescence, the adaxial surface glabrous, the tips and margins with a few sessile echinoid trichomes. Filament tube 0.5–1 mm long, the free portion of the filaments 1–1.5 mm long, glabrous, with tiny projections ca. 0.5 mm long on edge of tube between each filament; anthers 3–4 mm long, 1–1.5 mm wide, ellipsoidal, loosely connivent, yellow, poricidal at the tips, the pores lengthening to slits with age. Ovary densely pubescent with multangulate to echinoid trichomes; style 9–9.5 mm long, densely pubescent along its entire length with multangulate to echinoid trichomes with 4–30 rays ca. 0.2 mm long; stigma capitate, the surface minutely papillose, bright green in fresh plants. Fruit a globose berry, 0.6–0.7 cm in diameter, green when ripe, the pericarp thin, not shiny, unevenly pubescent with multangulate or echinoid trichomes with rays of many varying lengths, appearing scurfy; fruiting pedicels 1–1.1 cm long, ca. 1.5 mm in diameter at the base, woody, more or less erect. Seeds 40–50 per berry, 0.9–1 mm long, 0.9–1 mm wide, flattened-reniform, reddish or golden brown, the surfaces minutely pitted, the testal cells elongate.

**Figure 4.**
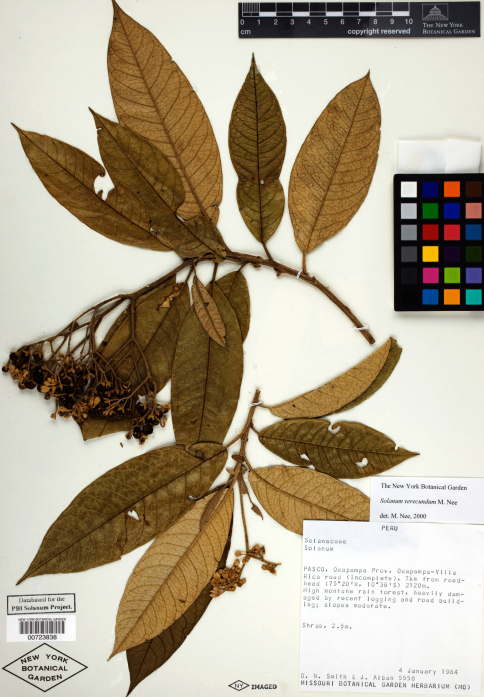
Isotype specimen of Solanum oxapampense S. Knapp. (Smith & Albán 5558 NY [NY00723838]). Specimen image reproduced with the permission of The C. V. Starr Virtual Herbarium of The New York Botanical Garden (http://sciweb.nybg.org/science2/VirtualHerbarium.asp).

##### Distribution.

Endemic to the valley of the Río Huancabamba (Río Pozuzo) in central Peru; found within the Parque Nacional Yanachaga-Chemillen (Fig. 2).

##### Ecology.

Montane forests from 1500 to 2500 m elevation; as with many members of the Brevantherum group, often found along roadsides and in open areas.

##### Etymology.

Named for the valley of Oxapampa, where this species is endemic.

##### Preliminary conservation status.

Solanum oxapampense is known from only 4 localities, has an EOO of 198 km2 and AOO (using a cell size of 0.04) of 195 km2; giving it a status of Endangered (IUCN 2001). If the IUCN (2001) recommended cell size is applied the AOO is reduced to 16 km2; the status remains Endangered.

##### Specimens examined.

**Peru:** Pasco: Prov. Oxapampa, Dist. Huancabamba, camino a Pozuzo, 1200–1400 m, 10°04.02S, 75°32.59W, 2 June 2004, R. Rojas, M. Huaman, A. Peña & J. Mateo 2490 (MO, NY [NY00824860], USM); Prov. Oxapampa, Dist. Oxapampa, Parque Nacional Yanachaga-Chemillen, Sector Chacos, 2471, 10°37'S, 75°17'W, 24 January 2004, R. Vasquez, A. Monteagudo, A. Peña & R. Francis 28939 (MO, NY[NY00829065], USM); Prov. Oxapampa, Dist. Oxapampa, Oxapampa-Villa Rica road, 2300 m, 10°39.59S, 75°19.33W, 22 November 2007, E. Tepe, S. Stern, J. Mateo, M.P. Moreno, R. Rojas 2227 (BM [BM001017349], USM, UT).

##### Discussion.

Solanum oxapampense is a very distinctive species with its coriaceous and strongly discolorous leaves, with the upper surfaces dark green and shiny and the veins deeply impressed, and the lower surfaces densely pubescent with pale tan peltate trichomes. It is similar to Solanum verecundum M. Nee with which it is broadly sympatric, but does not apparently co-occur. Solanum verecundum is found along the eastern slopes of the Andes from Ecuador to southern Peru, Solanum oxapampense is possibly derived from it. The two species share trichomes that are peltate to some degree, small white flowers and small fruits (orange in Solanum verecundum, apparently green at maturity in Solanum oxapampense). Solanum oxapampense differs from Solanum verecundum in its coriaceous, narrower leaves and in its pubescence, which is composed of truly peltate trichomes whose rays are fused for more than half their length and that lack midpoints. Trichomes of Solanum verecundum have rays that are only fused in the central half, near the midpoint; midpoints are always present, even if sometimes very small and nub-like.

In the original description of Solanum verecundum (Nee 2000), the plant illustrated in Figure 1 is Smith & Albán 5558 the type specimen selected here for Solanum oxapampense, not Jaramillo et al. 13285, the type specimen of Solanum verecundum as indicated in the text of Nee (2000). Smith and Albán 5558 is the only specimen of the species here recognised as Solanum oxapampense to have been included in the original circumscription of Solanum verecundum; an emended description of that species excluding this element is presented below and diagnostic characters separating the two are presented above.

Neither Solanum oxapampense nor Solanum verecundum would have been included in section Brevantherum by Roe (1972) due to their possession of peltate trichomes, despite the overall habit and inflorescence morphology. Nee (2000) mentioned this in the original description of Solanum verecundum (see below). Molecular data show these groups (the traditional sections Brevantherum and Lepidota (Dunal) Seithe) to be members of the same clade, defined by having ellipsoid anthers and variations on stellate trichomes (some of which have lost the rays altogether, e.g., section Gonatotrichum Bitter, see Stern and Bohs 2009).

#### Solanum verecundum

M.Nee

urn:lsid:ipni.org:names:1016242-1

Fig. 5 

##### Type.

**Ecuador:** Sucumbios: El Salado, colecciones en el sendero a la finca del Sr. Segundo Pacheco, 1400 m, 13 October 1990, J. Jaramillo, E. Grijalva & M. Grijalva 13285 (holotype: QCA; isotype: NY! [NY00381798]).

##### Description.

Shrub to small tree, 4–14 m tall. Stems densely pubescent with persistent short-stalked porrect –stellate to somewhat peltate trichomes 0.2–0.3 mm in diameter, the rays 10–12, fused for less than half their length, the midpoint sometimes a short stub to 0.5 mm long; new growth densely pubescent with porrect-stellate trichomes like those of the stems, these drying pale golden-brown. Bark of older stems reddish gold from the persistent trichomes. Sympodial units plurifoliate, the branching dichasial. Leaves simple, 6–19 cm long, 2–10 cm wide, elliptic or narrowly elliptic, membranous or chartaceous, discolorous (“silvery beneath" fide Bohs 3361), the upper surfaces moderately and evenly pubescent with sessile and short-stalked porrect-stellate trichomes with up to 15 rays, the rays fused only in their lower part near the midpoint, the midpoint to 0.2 mm long, the lamina visible, the lower surfaces densely pubescent with short-stalked porrect-stellate trichomes to 0.4 mm in diameter with up to 16 rays, the rays fused only in the center, the midpoint to 0.05 mm long, the lamina not visible; primary veins 12–15 pairs, not markedly impressed above, densely covered by pubescence beneath; base acute to somewhat attenuate onto the petiole; margins entire, plane; apex acute to acuminate; petioles 1–1–3(–4) cm long, densely pubescent with porrect-stellate to peltate trichomes like those of the stems. Inflorescences terminal, 7–10 cm long, many times branched, with 100+ flowers, densely pubescent with porrect-stellate to peltate trichomes like those of the stems; peduncle 2–5 cm long; pedicels 5–6 mm long, 1–1.5 mm in diameter at the base, ca.1.5 mm in diameter at the apex, stout, nodding at anthesis, densely pubescent like the inflorescence axes, articulated at the base; pedicel scars closely and more or less regularly spaced ca. 1 mm apart. Buds globose, the corolla strongly exserted from the calyx tube just before anthesis. Flowers all perfect, 5-merous. Calyx tube 1–1.5 mm long, cup-shaped, narrowing gradually to the pedicel, the lobes 1–1.5 mm long, deltate, abaxially densely pubescent with porrect-stellate to slightly peltate trichomes like those of the inflorescence, the adaxial surface sparsely pubescent with sessile porrect-stellate trichomes. Corolla 1–1.2 cm in diameter, white, stellate, lobed ca. ¾ of the way to the base, the lobes 4–5 mm long, 2–2.5 mm wide, reflexed at anthesis, the tips and margins densely pubescent on the abaxial surface with porrect-stellate trichomes with ca. 10 rays like those of the inflorescence, the adaxial surface glabrous, the tips and margins with a few sessile porrect-stellate trichomes. Filament tube minute, the free portion of the filaments ca. 1 mm long, glabrous; anthers 2.5–3 mm long, ca. 1 mm wide, ellipsoidal, loosely connivent, yellow, poricidal at the tips, the pores lengthening to slits with age. Ovary densely pubescent with multangulate to porrect-stellate trichomes; style 6–6.5 mm long, densely pubescent along its entire length with porrect-stellate 4–6-rayed trichomes ca. 0.2 mm long, the midpoints elongate and equal to the rays; stigma capitate, the surface minutely papillose. Fruit a globose berry, 0.5–1 cm in diameter, bright orange when ripe, the pericarp thin, not shiny, unevenly pubescent with multangulate trichomes with rays of many varying lengths, appearing scurfy; fruiting pedicels 0.9–1 cm long, 1.5–2 mm in diameter at the base, woody, erect. Seeds >100 per berry, 1–1.5 mm long, 1–1.5 mm wide, flattened-reniform, pale golden-yellow, the surfaces minutely pitted, the testal cells square.

**Figure 5.**
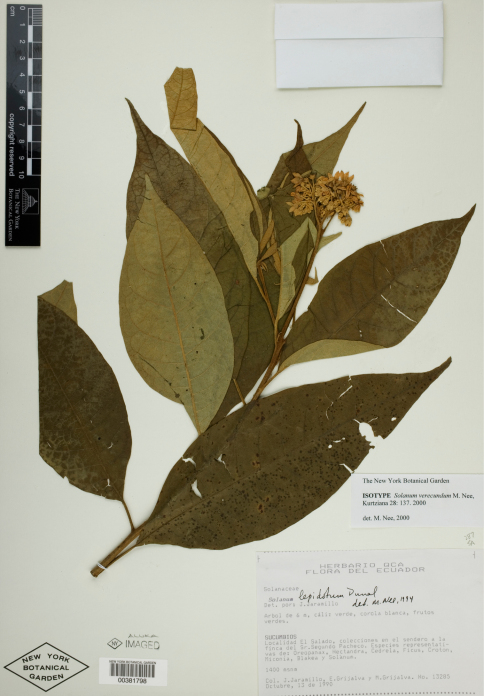
Isotype specimen of Solanum verecundum M.Nee. (Jaramillo et al. 13258, NY [NY00381798]). Specimen image reproduced with the permission of The C. V. Starr Virtual Herbarium of The New York Botanical Garden (http://sciweb.nybg.org/science2/VirtualHerbarium.asp).

##### Distribution.

Along the eastern slopes of the Andes from northern Ecuador to southern Peru (to the Department of Cuzco; see Nee, 2000 for a distribution map).

##### Ecology.

Solanum verecundum occurs in premontane and montane forests, primarily in secondary growth (“purma") and along roads, from 1200–2000 m.

##### Preliminary conservation status.

Solanum verecundum is a relatively common species with a broad distribution along the eastern slopes of the Andes and can be considered of Least Concern (IUCN 2001).

##### Specimens examined.

**Ecuador:** Napo: road Baeza-Tena, 8 km from Baeza, towards Río Cosanga, 1800–1900 m, 0°31'S, 77°50'W, 28 October 1976, H. Balslev & E. Madsen 10400 (BM [BM000935121]). Zamora-Chinchipe: road between El Progreso and Guaramizal, ca. halfway to Guaramizal, 1430 m, 4°48.23S, 79°07.26W, 28 March 2005, L. Bohs, J.L. Clark, J.R. Bennett, N. León et al. 3324 (BM [BM000846206]); Cantón Chinchipe, Parroquia Zumba, trail from Guaramizal to cabin of Sandy León, W of Escuela Byron Jiménez, just S of Las Pircas, 1600 m, 4°46.21S, 79°10.36W, 30 March 2005, L. Bohs, J.L. Clark, J.R. Bennett, N. León et al. 3361 (BM [BM000846204]); Cantón Valladolid, Parroquia Valladolid, road between Valladolid and El Porvenir del Carmen, close to Tapala, 1600–1680 m, 4°32.45S, 79°06.08W, 1 April 2005, L. Bohs, J.L. Clark, J.R. Bennett &N. León 3381 (BM [BM000846226]); Palanda, region de la Cordillera del Condor, Parroquia San Francisco de Vergel, riberas del Río Vergel, entre Santa Rosa y La Canela, 1200 m, 4°39.07S, 79°01.41W, 6 March 2007, W. Quizhpe & A. Wisum 2492 (BM [BM000943415]). Peru. Cajamarca: Prov. San Ignacio, San José de Lourdes, entre Camaná y Santo Tomás, 1800–1870 m, 5°01.00S, 78°52'W, 8 April 1997, J. Campos & S. Corrales 3801 (BM [BM000846199]); Prov. San Ignacio, San José de Lourdes, caserio Rumichina, 1679 m, 5°49.09S, 78°17.04W, 30 June 2006, J. Perea & V. Flores 2508 (BM [BM000943431]). Huánuco: Prov. Ambo, 38 km from Tingo Maria, between Tingo Maria and Pucallpa, 1680 m, 4 August 1978, J. Aronson & P. Berry 618 (BM [BM000795484]).

##### Discussion.

As mentioned above, the original description of Solanum verecundum (Nee 2000) included one specimen here segregated as Solanum oxapampense. The taxa differ in a suite of characters, detailed above in the discussion of Solanum oxapampense, but are easy to distinguish by leaf morphology; the leaves of Solanum oxapampense are coriaceous and shiny above while those of Solanum verecundum are membranous or chartaceous and pubescent above. These stellate trichomes cause the leaves to be asperous to the touch on dry specimens. The stellate trichomes of Solanum verecundum, while somewhat peltate like those of Solanum oxapampense, never have the rays fused for more than half their length, and always bear midpoints, even if these are quite tiny. Nee (2000) pointed out the morphological similarity between Solanum verecundum and the similarly widespread Solanum lepidotum Dunal and Solanum schlechtendalianum Walp. The latter two taxa have more lateral inflorescences that are not borne on erect peduncles. Solanum lepidotum and Solanum schlechtendalianum show a similar pattern of hair diversity to Solanum verecundum and Solanum oxapampense; each species pair has one member with porrect-stellate trichomes with the rays not fused (Solanum schlechtendalianum and Solanum verecundum) and one with peltate trichomes (Solanum lepidotum and Solanum oxapampense). Taxonomists working with primarily morphological data have traditionally recognised different sub-groupings for taxa with stellate and peltate trichomes (Seithe 1962, Carvalho 1996, Nee 1999), but molecular data (Bohs 2005, Weese and Bohs 2007) suggest a more complex situation.

## Supplementary Material

XML Treatment for Solanum kulliwaita

XML Treatment for Solanum dillonii

XML Treatment for Solanum oxapampense

XML Treatment for Solanum verecundum
